# COVID-19 Associated Coagulopathy in the Setting of Underlying Malignancy

**DOI:** 10.7759/cureus.10095

**Published:** 2020-08-28

**Authors:** Su Lin Lim, Kok Hoe Chan, Jihad Slim, Gunwant Guron, Hamid S Shaaban

**Affiliations:** 1 Internal Medicine, Saint Michael's Medical Center, Newark, USA; 2 Infectious Diseases, Saint Michael's Medical Center, Newark, USA; 3 Hematology and Oncology, Saint Michael's Medical Center, Newark, USA

**Keywords:** covid-19 associated coagulopathy, pulmonary embolism, deep venous thrombosis, metastatic ovarian cancer

## Abstract

Coronavirus disease 2019 (COVID-19) associated coagulopathy is a well-recognized predictor for morbidity and mortality in COVID-19 patients. Both deep vein thrombosis (DVT) and pulmonary embolism (PE) have been reported in COVID-19 patients. Nonetheless, there are no consensus guidelines on the use of therapeutic coagulation in this group of patients. We herein present a unique case of a confirmed COVID-19 patient with metastatic ovarian cancer who presented with DVT and PE despite being on therapeutic anticoagulation, highlighting the unpredictability of COVID-19 associated coagulopathy. This case study raises the awareness that the thrombophilic state in metastatic malignancies is potentially augmented by COVID-19. We also discuss the complexity of making anticoagulation treatment decision in COVID-19 patients in the absence of evidence-based guidelines.

## Introduction

Coronavirus disease 2019 (COVID-19) has reached a global public health emergency with more than 17 million cases and 680,000 deaths reported worldwide as of August 2, 2020 [1]. COVID-19 has multifaceted presentations with close to 70-80% of patients who are asymptomatic or mildly symptomatic [2]. COVID-19 associated coagulopathy is growingly recognized as the predictor for morbidity and mortality in COVID-19 patients [3,4]. To our knowledge, there are still no consensus guidelines on the management of this group of patients, including the choice of anticoagulation, the specific cut-off for D-dimer, fibrinogen, or coagulation markers, the duration of treatment, and follow-up recommendations. The majority of the data in the literature are on severe COVID-19 patients with exuberant inflammatory response associated with complications related to cytokine storm or hyperinflammatory phase, but there are not much data on asymptomatic or mild disease, which comprises the majority of the patients. We herein report a case of a 60-year-old female with metastatic ovarian cancer who tested positive for COVID-19 reverse transcriptase-polymerase chain reaction (RT-PCR) and developed deep vein thrombosis (DVT) and pulmonary embolism (PE) on day 15 of her illness despite being on therapeutic anticoagulation with apixaban 5 mg twice daily as outpatient and low molecular weight heparin (LMWH) as an inpatient. This highlights the importance of aggressive therapeutic anticoagulation in COVID-19 patients with active malignancy and further raises the discussion on the choice of anticoagulation in this group of patients.

A preprint of this manuscript was submitted to ResearchSquare: Lim, SL, Chan, KH, Guron, G and Shaaban, HS. COVID-19 Associated Coagulopathy in the Setting of Underlying Malignancy. ResearchSquare. May 28, 2020. doi: 10.21203/rs.3.rs-31264/v1

## Case presentation

**Figure 1:**
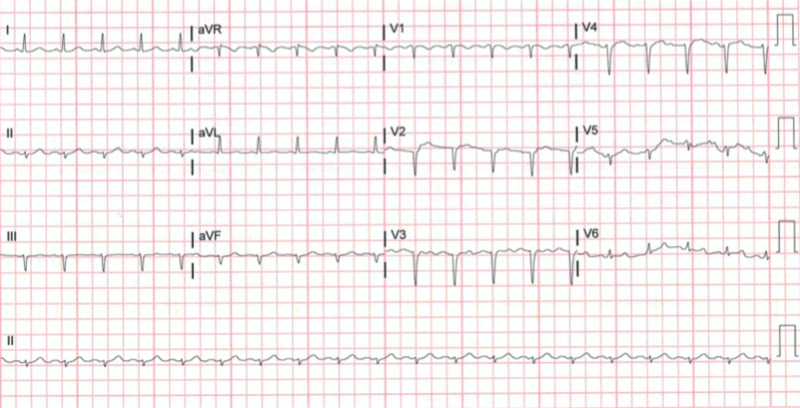
EKG showing sinus tachycardia with left axis deviation EKG, electrocardiogram

This is a case of a 60-year-old female with a past medical history of DVT and PE diagnosed eight months ago who was on apixaban 5 mg twice daily. She was newly diagnosed with metastatic ovarian cancer with peritoneal carcinomatosis and tested positive for COVID-19 on April 22, 2020, revealing that she had only minimal respiratory symptoms at that time, with good oxygen saturation. She was readmitted on April 28, 2020, two days after discharge, and presented with failure to thrive, constipation, and abdominal distention. Initial vital signs on admission showed temperature of 98.2°F, blood pressure of 123/86 mmHg, tachycardia with heart rate of 121 beats/min, and normal respiratory rate of 18 breaths/min, and she was saturating 99% on room air. Her body mass index was 22.3 kg/m^2^. On physical examination, she had decreased air entry to the lungs bilaterally with diffuse rhonchi and crackles, tense and distended abdomen with fluid shift, and 2+ bilateral pitting edema. Complete blood count showed WBC of 8.3 x 10^3^/uL (4-11 x 10^3^/uL), platelets of 359 x 10^3^/uL (150-450 x 10^3^/uL), partial thromboplastin time of 33.7 s (25.2-37.4 s), prothrombin time (PT) of 14.4 s (9.9-13.0 s), and procalcitonin of 0.1 ng/mL (0-0.5 ng/mL). Electrocardiogram (EKG) showed sinus tachycardia with left axis deviation (Figure 1).

**Figure 2:**
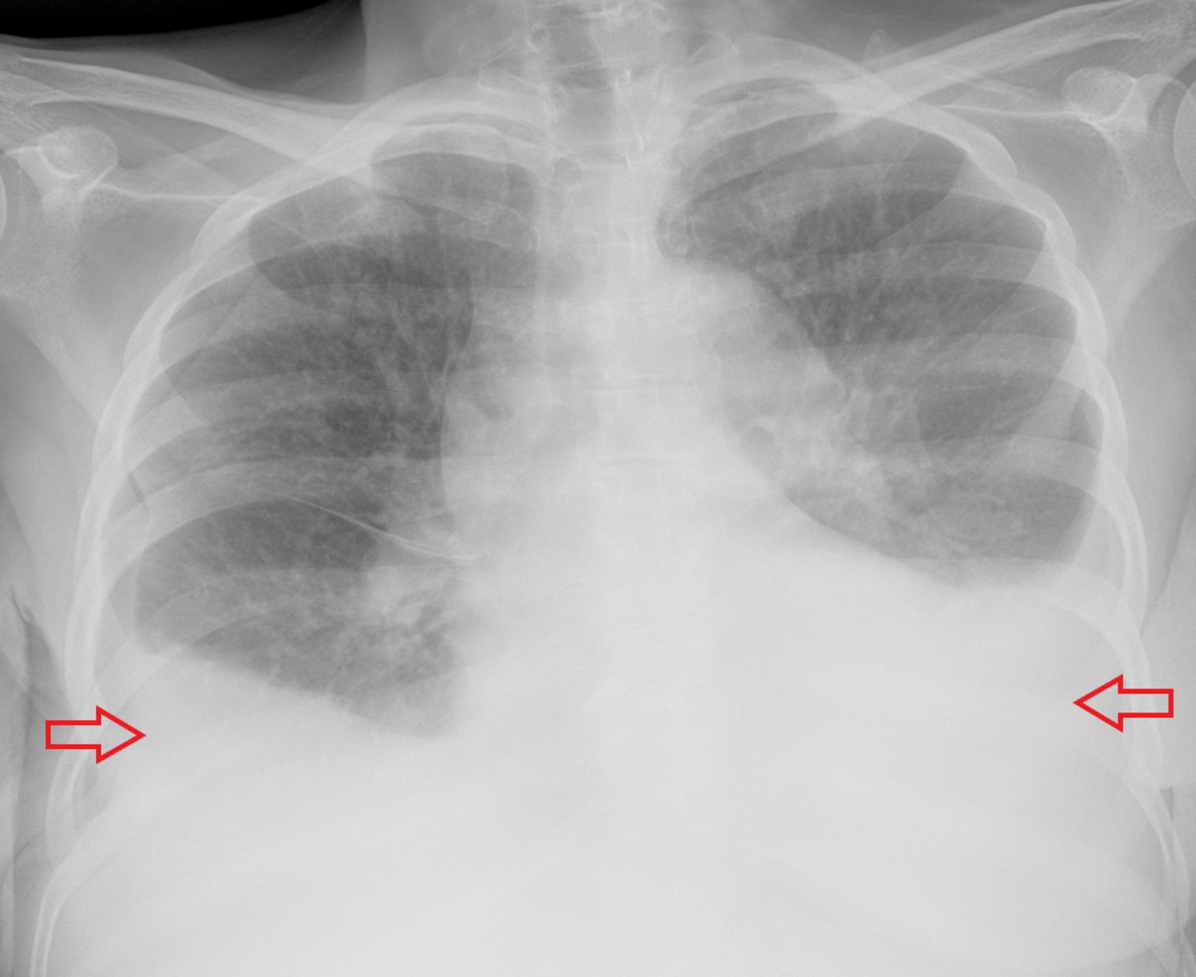
Chest X-ray showing bilateral pleural effusion with bibasilar atelectasis

Chest X-ray showed bilateral pleural effusion with bibasilar atelectasis (Figure 2).

**Figure 3:**
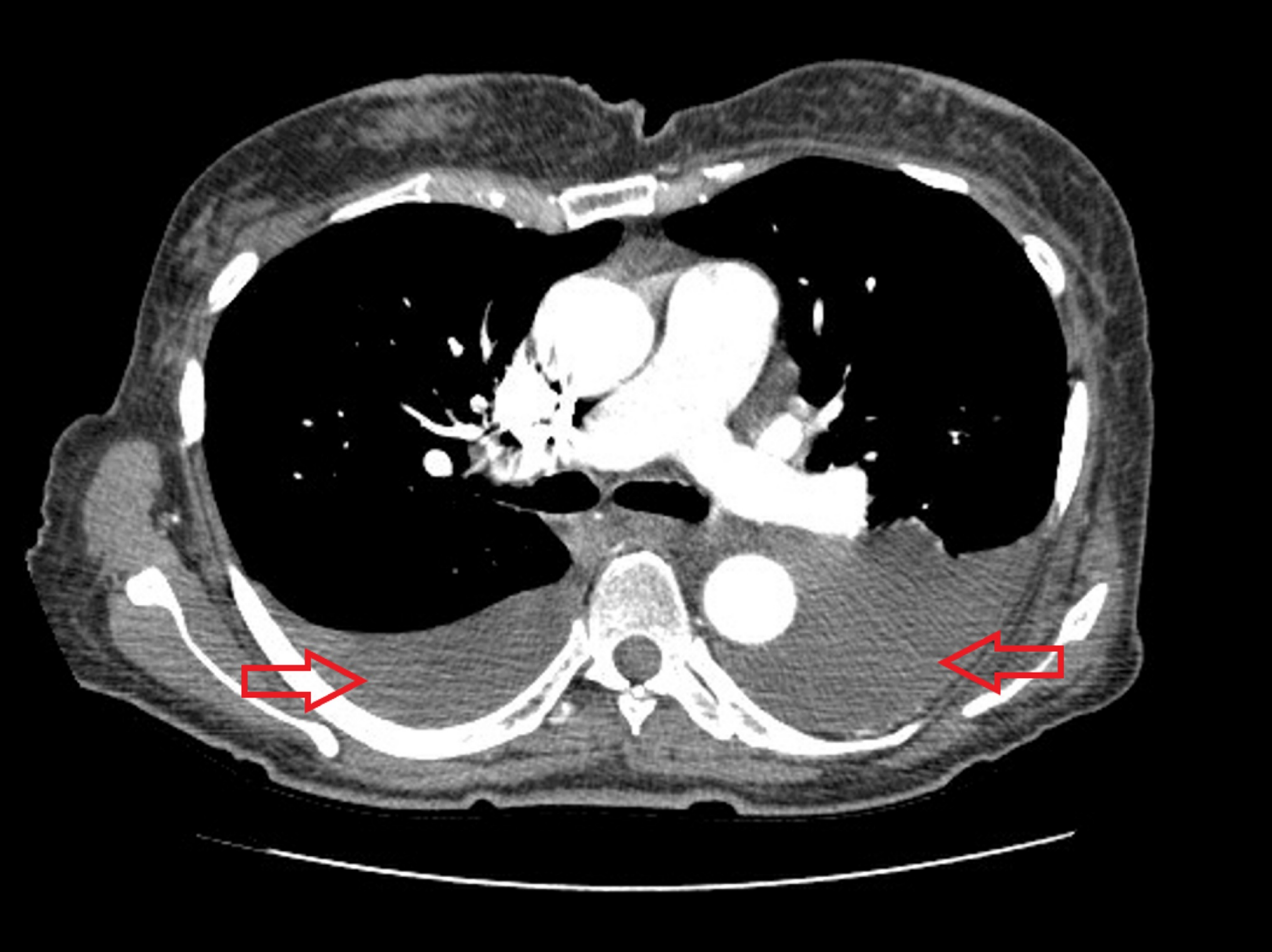
CTA performed on previous admission (April 20, 2020) showing no evidence of pulmonary embolism but bilateral pleural effusion (red arrows) CTA, computed tomography angiogram

Her computed tomography angiogram (CTA) performed on previous admission (April 20, 2020) showed no evidence of PE (Figure 3), with moderate bilateral pleural effusion, multiple pulmonary nodules, and 15-mm right pleural mass, which may represent metastatic disease with axillary and hilar lymphadenopathy.

**Figure 4:**
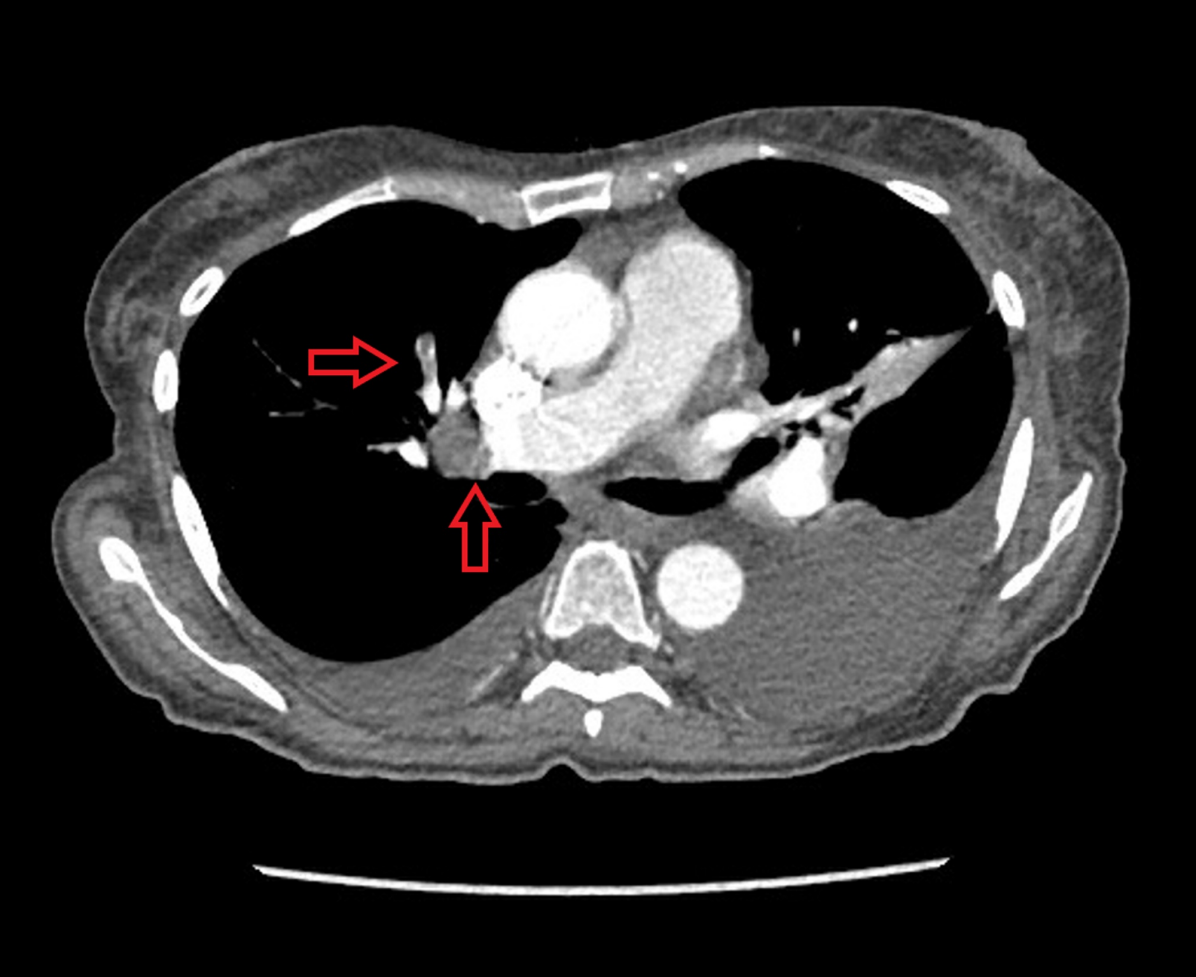
CTA of the chest showed pulmonary emboli throughout the right lung, with the greatest clot burden at the right middle lobe (red arrows) CTA, computed tomography angiogram

Thoracentesis was performed, which showed metastatic carcinoma immunophenotypically consistent with ovarian origin. Her inflammatory markers on last admission were as follows: D-dimer of 5,329 ng/mL (0-500 ng/mL), fibrinogen of 530 mg/dL (200-393 mg/dL), lactate dehydrogenase (LDH) of 569 U/L (122-222 U/L), C-reactive protein (CRP) of 17.5 mg/dL (0-0.8 mg/dL), and ferritin of 829.3 ng/mL (11-307 ng/mL). D-dimer decreased to 1,859 ng/mL on present admission, ferritin to 852.8 ng/mL, LDH to 587 U/L, and CRP to 13.8 mg/dL. She received therapeutic enoxaparin 60 mg every 12 hours during the hospital course in light of a history of DVT and PE. She was doing well, and inflammatory markers were trending down. On day 8 of admission (day 15 of COVID-19 symptoms), D-dimer was trending up to 7,786 ng/mL. In addition, she has persistent tachycardia with increased oxygen requirement. Although she was already on anticoagulation, but in light of multiple comorbidities and COVID-19 associated coagulopathy, repeat CTA of the chest was performed, which showed pulmonary emboli throughout the right lung, with the greatest clot burden at the right middle lobe (Figure 4), in addition to wedge-shaped opacities at the right middle lobe, which may represent developing pulmonary infarction.

CTA also showed ground-glass opacities at the peripheral and basal region, predominantly on the right lung. Venous Doppler was performed, which showed acute DVT in both lower extremities. Echocardiogram showed left ventricular ejection fraction (LVEF) of 50-55%, right ventricle moderately hypokinetic, moderate pulmonary hypertension, and interventricular septal flattening, suggestive of right ventricular strain from PE. Thrombolytic or catheter-directed thrombolytic therapy was not considered in this setting as the patient was hemodynamically stable. Inferior vena cava filter was placed and she was discharged to a subacute rehab center with apixaban and will continue palliative chemotherapy on carboplatinum and paclitaxel as an outpatient.

## Discussion

Venous thromboembolism (VTE) is common in acutely ill COVID-19 patients despite the use of prophylactic anticoagulation. It was reported that around 20-43% of VTE, mostly PE, developed in intensive care unit (ICU) patients [5-7]. Data are limited for inpatients admitted to the regular medical floor. The pathophysiology of COVID-19 associated coagulopathy remains unclear. Two autopsy studies on post-mortem examination of COVID-19 individuals revealed that common causes of death are hypercoagulability and inflammation [8,9]. One autopsy study revealed that 7 (58%) out of 12 patients had DVT and PE and this was the direct cause of death in four patients [9]. Here, we reported a case of asymptomatic COVID-19 patient with underlying malignancy and a previous history of VTE who developed an acute VTE (both DVT and PE) despite being on therapeutic anticoagulation. Initially, the patient’s D-dimer was trending down to 1,859 ng/mL, and later on, there was an increasing trend of D-dimer to 7,786 ng/ml on day 8 of admission, which prompted the suspicion of acute VTE. The initial CTA was negative, which suggested that the previous PE from eight months earlier had resolved.

Although this patient has multiple risk factors for VTE, which included malignancy, immobility, and a previous history of VTE, the inflammation and severe endothelial dysfunction secondary to COVID-19 may trigger and worsen the hypercoagulable state despite the use of therapeutic dose of enoxaparin. Interestingly, the late presentation of VTE in COVID-19 patients also suggests that the hypercoagulable state persists even in the recovery phase and also in asymptomatic patients. High D-dimer is an independent predictor of mortality in hospitalized patients with COVID-19 [10]. However, it is also important to trend D-dimer in asymptomatic COVID-19 patients in the hospital.

A recent study from Mount Sinai Hospital showed that therapeutic dose anticoagulation improved survival among hospitalized COVID-19 patients both in and out of ICU [11]. Due to a lack of data, the use of therapeutic anticoagulation in individuals with no documented VTE still remains controversial. The American Society of Hematology (ASH) recommends the use of prophylactic anticoagulation for those who have not had confirmed VTE unless in patients with high-risk probability in whom confirmatory test could not be performed. If VTE is suspected, confirmatory testing should be sought if possible. Despite the lack of evidence, many institutions have used an intermediate dose or therapeutic dose of anticoagulation on managing COVID-19 coagulopathy. Therefore, current clinical trials are focusing on studying the therapeutic or prophylactic dose of anticoagulation in preventing VTE in critically ill COVID-19 patients. It is also crucial to understand which phase of disease is associated with the highest risk of VTE to know the optimal timing and duration for therapeutic anticoagulation. It will be interesting to study the incidence of VTE in asymptomatic COVID-19 patients with high risk as well. In regard to the duration of anticoagulation, it is important to consider that extended thromboprophylaxis after discharge depends on a patient's VTE risk factors.

Maatman et al. and Ranucci et al. have recently described the distinct pattern of COVID-19 coagulopathy on thromboelastography or rotational thromboelastometry [12,13]. Nonetheless, there is no consensus and evidence to date on the application of this information in guiding the anticoagulation therapy. More studies are still needed to assess the potential role of using the viscoelastic testing in the future, especially in patients with malignancy and COVID-19 coagulopathy.

Heparins have been reported to have an anti-inflammatory and anti-viral activity in COVID-19. They bind tightly to COVID-19 spike proteins, downregulate interleukin-6, and directly dampen immune activation [14-16]. To date, there is no study that compares whether unfractionated heparin (UFH) is better than LMWH in COVID-19 coagulopathy. Koenig et al. reported that heparin, in addition to anticoagulation, has effects on the blocking of P- and L-selectin, which are being reduced or even eliminated by the switch to the LMWHs [17]. Although there is no direct comparison between heparin and LMWH, it seems that heparins have more functional roles that have been reduced with conversion to LMWH and synthetic polysaccharides. Nonetheless, LMWH has more practical use due to less frequent dosing and omits the need for frequent monitoring of activated PTT (aPTT). However, UFH has better anti-inflammatory activity and is preferred in patients who may need reversibility for urgent intervention, morbid obesity, or renal impairment. The other indication to use UFH is in patients who progressed despite being on anticoagulant therapy. More studies are still needed to dissect the roles and differences of UFH versus LMWH on its anti-inflammatory activity, especially on COVID-19.

## Conclusions

In conclusion, we presented a case of COVID-19 infection that triggered the coagulation cascade in an asymptomatic high-risk patient despite being on therapeutic anticoagulation. It highlighted that the risk of VTE persisted until the late phase of disease. This shows the importance of early recognition of acute VTE with immediate treatment even in the recovery phase to decrease mortality. It also emphasizes the role of extended anticoagulation after discharge in the future management of COVID-19 patients who are at high risk of VTE.
